# Effect of GaN Cap Thickness on the DC Performance of AlGaN/GaN HEMTs

**DOI:** 10.3390/mi15050571

**Published:** 2024-04-26

**Authors:** Zuorong Nie, Kai Wang, Xiaoyi Liu, Hong Wang

**Affiliations:** 1 Engineering Research Center for Optoelectronics of Guangdong Province, School of Physics and Optoelectronics, South China University of Technology, Guangzhou 510640, China; 2 School of Electronics and Information Engineering, South China University of Technology, Guangzhou 510640, China; 3 Zhongshan Institute of Modern Industrial Technology, South China University of Technology, Zhongshan 528437, China

**Keywords:** GaN cap, HEMTs, hall effect, surface morphology, off-state characteristics

## Abstract

We prepared AlGaN/GaN high electron mobility transistors (HEMTs) with GaN cap thicknesses of 0, 1, 3, and 5 nm and compared the material characteristics and device performances. It was found that the surface morphology of the epitaxial layer was effectively improved after the introduction of the GaN cap layer. With the increase of the GaN cap thickness, the carrier concentration (n_s_) decreased and the carrier mobility (μ_H_) increased. Although the drain saturation current (I_dSat_) of the device decreased with the increasing GaN cap thickness, the excessively thin GaN layer was not suitable for the cap layer. The thicker GaN layer not only improved the surface topography of the epitaxial layer but also effectively improved the off-state characteristics of the device. The optimal cap thickness was determined to be 3 nm. With the introduction of the 3 nm GaN cap, the I_dSat_ was not significantly reduced. However, both the off-state gate leakage current (I_gLeak_) and the off-state leakage current (I_dLeak_) decreased by about two orders of magnitude, and the breakdown voltage (BV) increased by about 70 V.

## 1. Introduction

AlGaN/GaN HEMTs are extensively used in applications requiring high power and high frequency capabilities due to their wide band gap, high mobility, and low on-resistance [1,2,3,4]. Due to the high affinity of Al for O, it is common to introduce a GaN cap layer above the barrier layer to suppress the oxidation of the AlGaN. Numerous researchers have investigated the influence of the GaN cap on the performance of AlGaN/GaN HEMTs. Yu et al. obtained the relationship between the two-dimensional electron gas (2DEG) concentration and the thickness of each layer for GaN/AlGaN/GaN HEMTs by theoretical calculations, and they pointed out that the increase in the thickness of GaN led to a decrease in the 2DEG concentration [5]. Jurkovic et al. reported a case that negative polarization charges appeared at the upper interface of the barrier layer when introducing a GaN cap layer above the InAlN/GaN heterojunction, leading to a significant decrease in 2DEG concentration [6]. Recently, researchers have discovered that the introduction of a GaN cap layer can effectively passivate the surface of an HEMT, reducing the density of surface defect states. Their efforts in utilizing the GaN cap layer to suppress off-state leakage current and current collapse in the devices have yielded significant results [7,8,9,10,11,12]. Furthermore, researchers have achieved commendable results by using density-functional theory (DFT) to study GaN-based epitaxial layers, which can help to adjust the epitaxial layer growth process to optimize the growth quality and reduce the interfacial states [13,14].

The selection of the thickness of the GaN cap is always a challenge. Variations in the thickness of the GaN cap affect the various properties of the device to varying degrees. Devices for different application scenarios require different performance parameters. Therefore, studies on the effect of GaN cap thickness on HEMTs are essential.

In this study, the impact of GaN cap thickness on the performance of AlGaN/GaN HEMTs has been analyzed. It was found that an increase in GaN cap thickness led to a decrease in surface roughness. As the cap thickness increased, the n_s_ decreased, while the μ_H_ increased. The increase in GaN cap thickness within 0–3 nm did not significantly degrade the I_dSat_ of the device, which was attributed to the significantly increased μ_H_. Additionally, it was found that the increase in cap thickness led to a decrease in I_gLeak_ and an increase in BV, improving the off-state performance of the device.

## 2. Experimental Details

All the samples obtained in this work were grown on 6-inch-high resistance Si (111) substrates by metal organic vapor deposition (MOCVD). TMGa, TMAl, and NH_3_ were used as precursors for Ga, Al, and N sources, respectively. H_2_ and N_2_ were used as carrier gases. Deposition started with a 150 nm AlN nucleation layer followed by a 400 nm-thick Al_0.25_Ga_0.75_N layer. Subsequently, a 640 nm Fe-doped GaN layer was grown at a doping concentration of 2.19 × 10^18^ cm^−3^ to improve the off-state characteristics of the device. The 760 nm unintentionally doped GaN layer was then grown. Then a 1 nm AlN insertion layer, a 22 nm Al_0.25_Ga_0.75_N barrier layer, and an optional GaN cap were incorporated. The cap thicknesses of the four samples were 0, 1, 3, and 5 nm, respectively. After growth, the surface morphology of all these samples was measured using atomic force microscopy (AFM) with the scan area of 5 × 5 μm^2^. Hall effect measurements were carried out on these samples at 300 K using the van der Pauw configuration.

**Figure 1:**
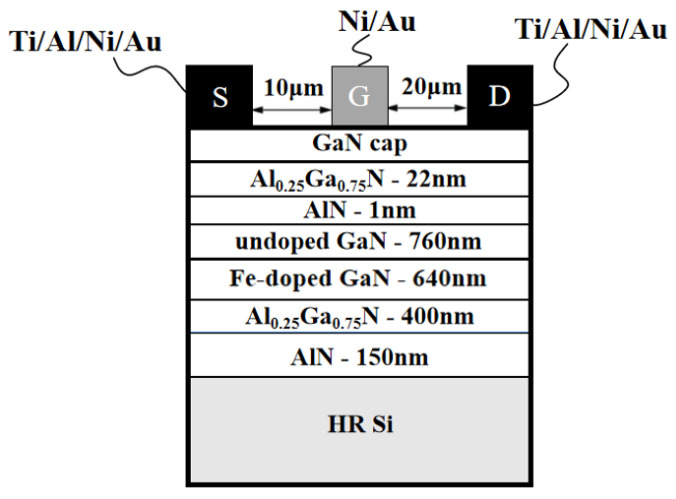
Schematic cross-section of HEMTs.

After growth, by employing inductively coupled plasma etching (ICP) to perform dry etching all the way down to the HEMT structure, device mesa isolation was achieved. Subsequently, the samples were immersed in buffered oxide etchant (BOE) for 60 s to remove the surface oxides. Ohmic contact was made by the deposition of Ti/Al/Ni/Au (20/100/10/100 nm), and the samples were subsequently annealed at 790 °C for 30 s in N_2_ atmosphere. The gate electrode was composed of Ni/Au (50/200 nm), which was fabricated by electron beam evaporation. Furthermore, the device dimensions used for these studies were as follows: L_G_/W_G_/L_GS_/L_DS_ = 1/100/10/20 μm. The schematic of the devices is shown in Figure 1.

## 3. Results and Discussion

**Figure 2:**
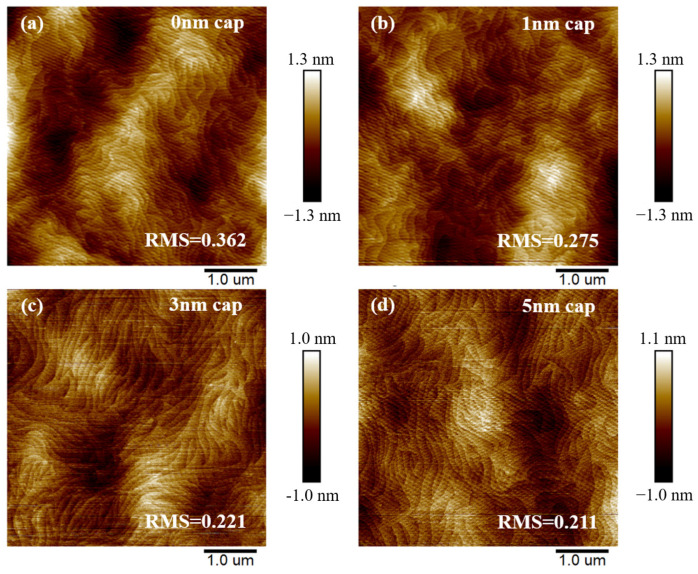
AFM image (scan area = 5 μm × 5 μm) of samples with cap thickness of (**a**) 0 nm, (**b**) 1 nm, (**c**) 3 nm, (**d**) 5 nm.

Figure 2a–d respectively show the AFM micrographs of samples with cap thickness of 0, 1, 3, and 5 nm. From Figure 2, the well-defined step flow morphology can be observed from the surfaces of these four samples. The obvious step flow morphology is the result of the high efficiency growth of the film. The root mean square (RMS) roughness of the sample surface was significantly reduced by the introduction of GaN. Similar observations have been documented in many studies [7]. A GaN cap layer can inhibit oxidation on the surface of the epitaxial layer, which may account for the reduced surface roughness. It was observed that as the cap thickness increased from 1 nm to 5 nm, the RMS of the wafer decreased from 0.275 nm to 0.211 nm, making the surface of the film even smoother. However, the surface roughness of a sample with a 5 nm cap is comparable to that of the sample with a 3 nm cap. We speculate that the protrusions on rough surfaces may hinder the lateral migration of Ga, leading to a tendency for GaN to form in the depressions, thus reducing the surface roughness of the film [15,16,17].

**Figure 3:**
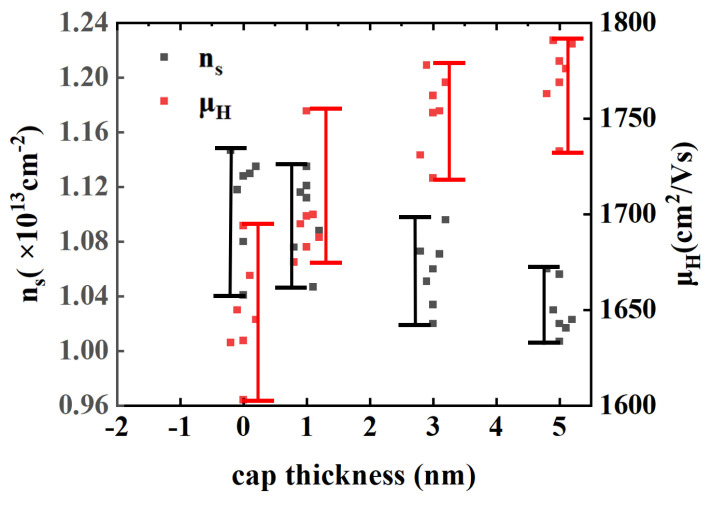
The room temperature Hall test results for samples with cap thickness of 0, 1, 3, and 5 nm.

Figure 3 shows the values of 2DEG mobility (μ_H_), 2DEG density (n_s_), and sheet resistance (R_s_) of samples with cap thicknesses of 0, 1, 3, and 5 nm measured at 300 K. As the cap thickness increased from 0 nm to 5 nm, the n_s_ decreased from 1.13 × 10^13^ cm^−2^ to 1.02 × 10^13^ cm^−2^. Due to the lattice mismatch between the GaN cap layer and the AlGaN barrier layer, the increase in cap thickness led to an enhanced piezoelectric polarization field above the barrier layer. This raised the conduction band and decreased the n_s_. As the cap thickness increased, the μ_H_ increased. The decrease in n_s_ led to a reduction in the intensity of roughness scattering at the heterojunction interface [18,19]. In addition, as the cap thickness increased, the surface roughness of these samples decreased and the distance from the 2DEG to the surface of the epitaxial layer increased, which may lead to a decrease in remote scattering intensity from the surface charges [20,21]. In addition, the difference in μ_H_ between the sample with a 3 nm cap and the sample with a 5 nm cap was observed to be nonsignificant. We speculate that the lower n_s_ led to a reduced effect of the n_s_ change on the roughness scattering intensity of the heterogeneous interface. As the cap thickness increased, interface roughness scattering intensity and the remote scattering intensity from the surface charges decreased, which made the effect of both on the μ_H_ decrease. Therefore, the increase in mobility was no longer significant.

**Figure 4:**
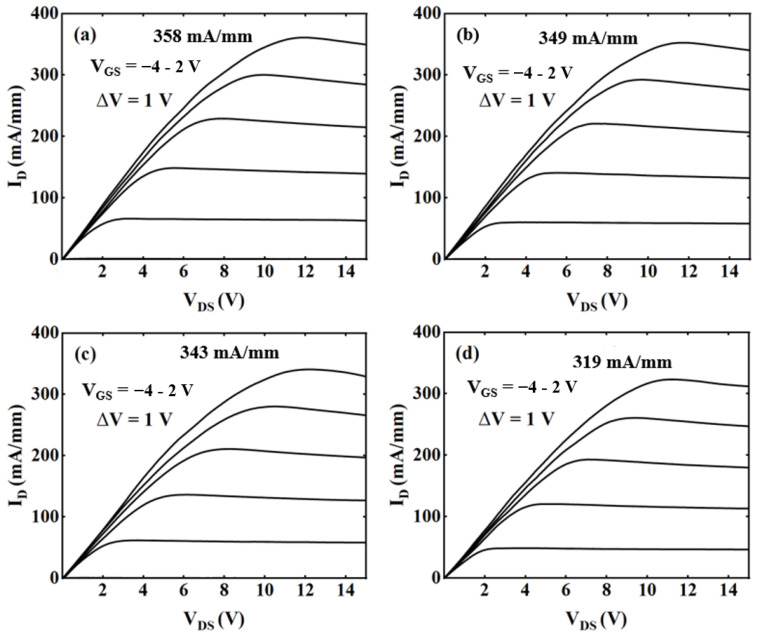
Output characteristics of samples with cap thickness of (**a**) 0 nm, (**b**) 1 nm, (**c**) 3 nm, and (**d**) 5 nm.

Figure 4 shows the output characteristic curves of these four samples. The I_dSat_ of the devices with cap thickness of 0, 1, 3, and 5 nm are 358 mA/mm, 349 mA/mm, 343 mA/mm, and 319 mA/mm, respectively, at a gate-to-source voltage (V_GS_) of 2 V. As the cap thickness increased, the I_dSat_ of the device decreased. However, as the cap thickness increased from 0 to 3 nm, the change in I_dSat_ of the device was not significant. Although the 2DEG concentration decreased with the increase in cap thickness, a significant increase in carrier mobility was observed.

**Figure 5:**
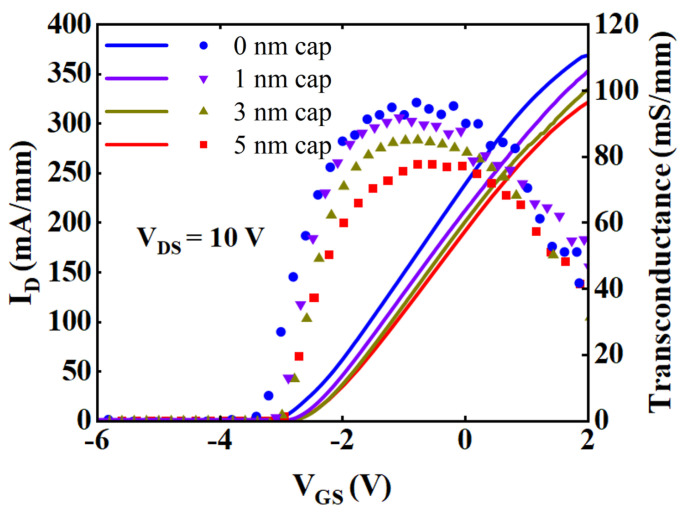
Transfer characteristics of samples with cap thickness of 0, 1, 3, and 5 nm.

The transfer characteristics of these four samples are depicted in Figure 5. The V_th_ is defined as the drain voltage at which the drain current is 1 mA/mm. The V_th_ of samples with cap thickness of 0, 1, 3, and 5 nm are −2.92 V, −2.86 V, −2.8 V, and −2.8 V respectively. The V_th_ is the point at which a device transitions from an on-state to an off-state. Although the distance between the gate and the channel layer increased with increasing GaN cap thickness, making it more difficult to deplete the carrier in the channel, the reduced carrier density compensates for this effect and minimizes the variation in the V_th_ of all these samples. Therefore, the disparity in V_th_ among the samples is slight. The transconductance (G_m_) of the devices with cap thicknesses of 0, 1, 3, and 5 nm are 96 mS/mm, 92 mS/mm, 86 mS/mm, and 77 mS/mm, respectively. The G_m_ is positively correlated with gate capacitance and carrier concentration. As the GaN cap thickness increased, the distance between the gate and the channel layer increased, resulting in a decrease in gate capacitance. Additionally, the decrease in n_s_ further led to the decrease in G_m_.

**Figure 6:**
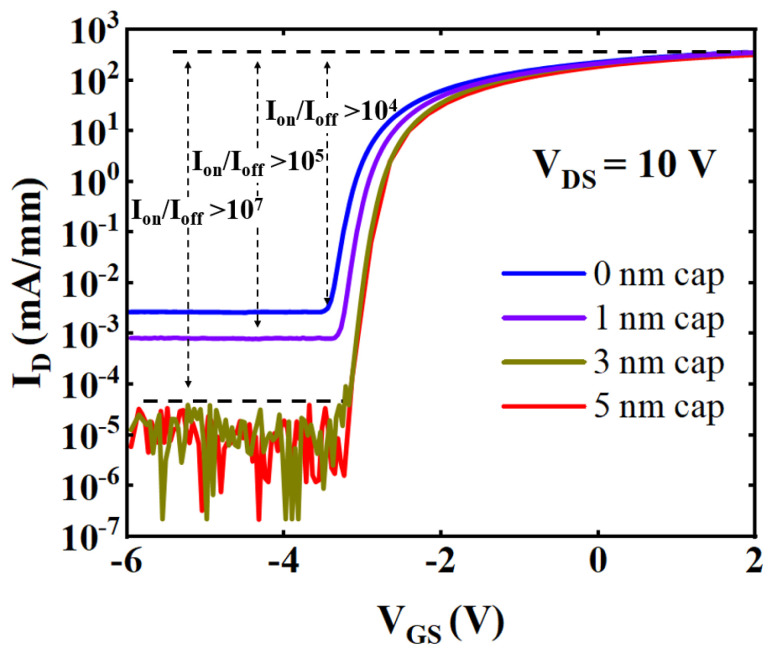
Transfer characteristics of samples with cap thicknesses of 0, 1, 3, and 5 nm with drain current on a log scale.

Figure 6 shows the drain current as a function of V_GS_ expressed on a log scale. It was observed that the I_dLeak_ of the device decreased as the cap thickness increased, and the I_dLeak_ of the device decreased by about two orders of magnitude after the cap thickness increased from 0 to 3 nm. The drain current ON/OFF (I_on_/I_off_) ratio of the devices with cap thickness of 3 nm and 5 nm were both higher than 10^7^ due to the reduced I_dLeak_. Thus, the increased GaN cap thickness reduced the I_dLeak_ and improved the I_on_/I_off_ ratio.

**Figure 7:**
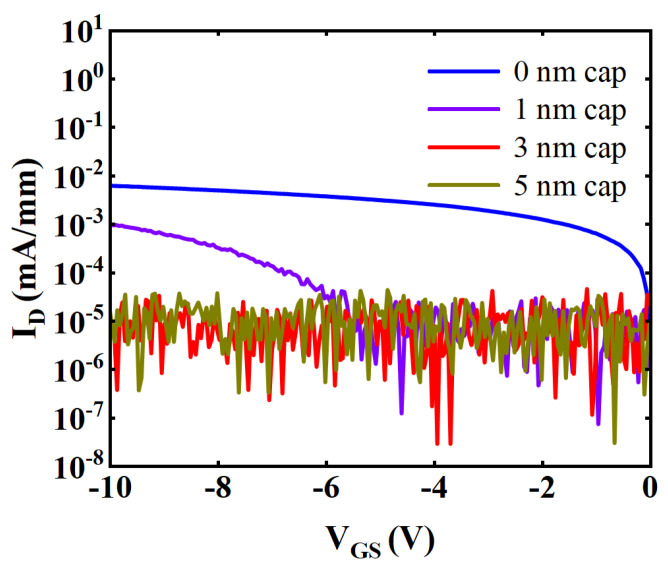
Off-state gate leakage characteristics of samples with cap thickness of 0 nm, 1 nm, 3 nm, and 5 nm.

**Figure 8:**
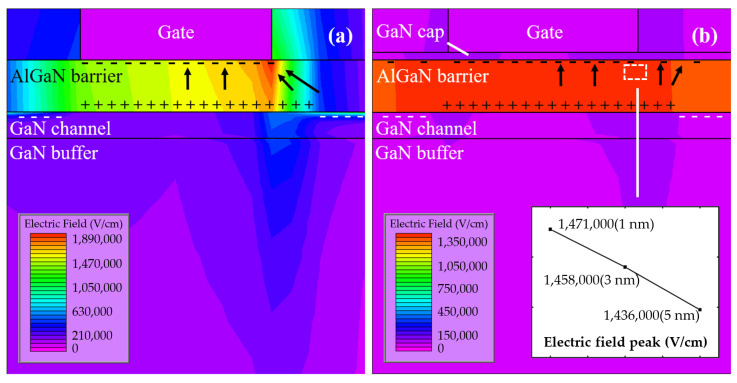
Simulated electric field profile under the gate at a biasing condition of V_GS_ = −8 V, and V_DS_ = 0 V, with embedded figure of peak electric field against cap thickness.

Figure 7 shows the gate leakage curves of samples with cap thickness of 0, 1, 3, and 5 nm. As the cap thickness increased, I_gLeak_ decreased. To appreciate the effect of GaN cap thickness on the off-state characteristics of the device, simulation was performed using Silvaco TCAD 2014. Figure 8 shows the electric field distribution of the device under a biasing condition of V_GS_ = −8 V, and V_DS_ = 0 V. Figure 8a shows the electric field distribution of the device without GaN cap layer, where the electric field is concentrated at the gate edge due to the lateral electric field crowd. As shown in Figure 8b, the introduced GaN cap layer homogenized the electric field in the barrier layer, leading to a decrease in peak electric field [22]. The peak electric field decreased as the thickness of the GaN cap layer increased. We speculate that the negatively polarized electric field introduced by the GaN cap layer reduced the potential at the top of the barrier layer. It increased the vertical electric field in the GaN barrier layer, thus reducing the lateral electric field crowding [22,23,24,25]. As the thickness of the GaN cap increased, the potential at the top of the barrier layer further decreased and the electric field distribution further homogenized. Therefore, the increase in GaN cap thickness can effectively reduce the peak electric field near the gate. As the GaN cap thickness increased, the electric field peak in the barrier layer decreased and the barrier thickness increased, which effectively suppressed the vertical tunneling of electrons and thus reduced the I_gLeak_. The thick GaN cap layer also reduced the carrier density under the gate and improved the barrier thickness, which further inhibited the vertical tunneling of electrons. In addition, the GaN cap layer could also inhibit the natural oxidation of the epitaxial layer. The reduction of O impurities on the surface of the epitaxial layer could reduce the hopping probability of electrons through the defect state and further reduce the I_gLeak_ [26,27,28].

**Figure 9:**
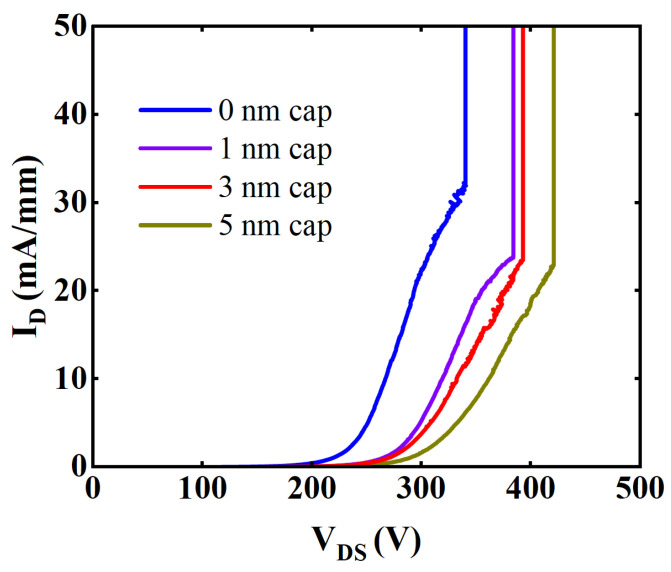
Breakdown characteristics of samples with cap thickness of 0, 1, 3, and 5 nm.

Figure 9 shows the breakdown characteristic curves of all these samples. It was observed that the BV of devices with cap thicknesses of 0, 1, 3, and 5 nm were 325 V, 383 V, 393 V, and 414 V respectively. As the cap thickness increased, the BV increased. As mentioned previously, the increase in GaN cap thickness inhibited the I_gLeak_ and the I_dLeak_, resulting in an increase in BV.

## 4. Conclusions

We have investigated the material and electrical properties of AlGaN/GaN HEMTs with GaN cap thicknesses of 0, 1, 3, and 5 nm, respectively. Although the increased cap thickness causes a reduced carrier concentration, a too-thin GaN cap layer is not suitable as a cap for an HEMT. The too-thin GaN cap leads to an increase in the surface roughness of the epitaxial layer and a degradation in device off-state performance. A too-thick GaN cap layer significantly reduces the I_dSat_ of the device. The optimized cap thickness was 3 nm. As the cap thickness increased from 0 to 3 nm, the surface roughness of the samples decreased from 0.362 nm to 0.221 nm. Meanwhile, I_dSat_ decreased by less than 10%, while both I_dLeak_ and I_gLeak_ decreased by about 2 orders of magnitude, and BV increased by about 70 V. The results for the quantification and reproducibility of HEMT clearly indicate the role of GaN cap thickness.

## Data Availability

The original contributions presented in the study are included in the article, further inquiries can be directed to the corresponding author.
